# 3D-Printed Bioactive Calcium Silicate/Poly-ε-Caprolactone Bioscaffolds Modified with Biomimetic Extracellular Matrices for Bone Regeneration

**DOI:** 10.3390/ijms20040942

**Published:** 2019-02-21

**Authors:** Yuan-Haw Andrew Wu, Yung-Cheng Chiu, Yen-Hong Lin, Chia-Che Ho, Ming-You Shie, Yi-Wen Chen

**Affiliations:** 1 School of Medicine, China Medical University, Taichung 40447, Taiwan; u102001503@cmu.edu.tw (Y.-H.A.W.); e0919908626@yahoo.com.tw (Y.-C.C.); 2 3D Printing Medical Research Center, China Medical University Hospital, Taichung 40447, Taiwan; roger.lin0204@gmail.com (Y.-H.L.); sfox1223@gmail.com (C.-C.H.); eric@mail.cmu.edu.tw (M.-Y.S.); 3 Department of Orthopedics, China Medical University Hospital, Taichung 40447, Taiwan; 4 The Ph.D. Program for Medical Engineering and Rehabilitation Science, China Medical University, Taichung 40447, Taiwan; 5 School of Dentistry, China Medical University, Taichung 40447, Taiwan; 6 Department of Bioinformatics and Medical Engineering, Asia University, Taichung 40447, Taiwan; 7 Graduate Institute of Biomedical Sciences, China Medical University, Taichung 40447, Taiwan; 8 3D Printing Medical Research Institute, Asia University, Taichung 40447, Taiwan

**Keywords:** calcium silicate, polycaprolactone, 3D scaffold, decellularized, extracellular matrix, osteogenesis

## Abstract

Currently, clinically available orthopedic implants are extremely biocompatible but they lack specific biological characteristics that allow for further interaction with surrounding tissues. The extracellular matrix (ECM)-coated scaffolds have received considerable interest for bone regeneration due to their ability in upregulating regenerative cellular behaviors. This study delves into the designing and fabrication of three-dimensional (3D)-printed scaffolds that were made out of calcium silicate (CS), polycaprolactone (PCL), and decellularized ECM (dECM) from MG63 cells, generating a promising bone tissue engineering strategy that revolves around the concept of enhancing osteogenesis by creating an osteoinductive microenvironment with osteogenesis-promoting dECM. We cultured MG63 on scaffolds to obtain a dECM-coated CS/PCL scaffold and further studied the biological performance of the dECM hybrid scaffolds. The results indicated that the dECM-coated CS/PCL scaffolds exhibited excellent biocompatibility and effectively enhanced cellular adhesion, proliferation, and differentiation of human Wharton’s Jelly mesenchymal stem cells by increasing the expression of osteogenic-related genes. They also presented anti-inflammatory characteristics by showing a decrease in the expression of tumor necrosis factor-alpha (TNF-α) and interleukin-1 (IL-1). Histological analysis of in vivo experiments presented excellent bone regenerative capabilities of the dECM-coated scaffold. Overall, our work presented a promising technique for producing bioscaffolds that can augment bone tissue regeneration in numerous aspects.

## 1. Introduction

When compared to wounds in other tissue types, hard tissue defect/wound regeneration is a relatively longer process. Chronic inflammation, impaired angiogenesis, and insufficient gene expression of growth factors in wound areas are some of the common causes of poor regeneration in tissue wounds [1]. The extracellular matrices (ECM) provide human cells with a complex cellular environment and maintain an ideal structure for cells at both the tissue and organ levels. ECMs intricately interact with cells and they ultimately regulate the extent of cellular migration, differentiation, and proliferation [2]. Stromal cells produce various ECM components, including different proteins and proteoglycans, which were complexed with each other in different ratios that distinctively characterize the unique composition, phenotypes, mechanical properties, and intrinsic biochemical pathways within a specific type of tissue or organ [3,4]. In fact, abnormalities in the properties of the ECMs may lead to loss of cellular homeostasis and functions.

Decellularization is a technique that has been increasingly employed in tissue engineering, because the resulting decellularized ECM (dECM) retains the native biological molecules that are instructive, as described above, for cell behavior and regulation, conferring a microenvironment that enhances cellular adhesion, proliferation, migration, and differentiation [5]. The dECM has been shown to reduce the effect and onset of inflammation. For example, Guilak et al. showed that decellularized ECM from biological tissues could significantly enhance wound healing, even in chronic-inflammation-prone diabetic rat models [6]. Furthermore, the removal of cellular components during decellularization reduces the properties that elicit immune responses; reduced immune responses have been observed in autologous, allogeneic, and xenogeneic decellularized scaffolds [7,8]. In our previous study, we demonstrated that ECM of MG63 cells contained collagen I (Col I), vitronectin, fibronectin (FN), and a number of other biological components, and it can assist stem cells in differentiating into mature osteogenic lineage cells [9]. This biologically significant ECM also provides a medium for cell-to-cell interaction with neighboring ECM [10]. Several studies have attempted to mimic the ECM microenvironment by integrating supporting molecules into synthetic biomaterials, such as hydrogels, ceramics, and synthetic polymers [11]; however, these methods only provide fragmental components that present specific functional receptors for cell attachment or proliferation and that cannot duplicate the complexity of natural ECM, which can, otherwise, be easily achieved through decellularization [5]. Hence, ECM has been recently frequently incorporated into bone tissue engineering. Published studies have recorded enhanced osteogenesis being demonstrated from constructs made from cell-derived ECM and PCL, titanium, or other synthetic biomaterials [12,13].

In bone tissue engineering, the scaffold designs have been extensively studied in an effort to achieve similarity with the complex three-dimensional structure of biological organs [14,15]. In a previous study, several kinds of organic–inorganic hybrid materials have been widely proposed, together with approaches to affect their physicochemical and biological properties [16,17]. Adequate porosity and sufficient strength are essential scaffold characteristics for bone tissue engineering. However, scaffolds that have intra-scaffold spaces, in which the pores are continuously linked, have been difficult to manufacture [18,19]. The continuity of porous spaces is critical for tissue regeneration, as it not only allows more tissue growth in the scaffold, but also encourages the development of uninterrupted vessel pathways during angiogenesis. Three-dimensional (3D) printing is a technique that can be used to manufacture 3D objects in a personalized manner with the help of computer-aided design [20]. It could, thus, play a revolutionary role in the making of scaffolds for tissue engineering, since this technique overcomes existing limitations by making the most suitable scaffolds for tissue regeneration through the simple and effective porosity dimension and design alterations that cannot be achieved through traditional scaffold manufacturing techniques [21,22]. The useful qualities of additive manufacturing technology could also be further implemented in bio-printing and bio-scaffolding of biological objects with intricate architectures [23,24].

Polycaprolactone (PCL) is a polymer that has excellent biocompatibility with low immunogenicity effects [25,26]; it has attracted a great deal of attention in the medical 3D-printing field, because the FDA-approved PCL can be easily manufactured into scaffolds of various 3D shapes due to its relatively low melting point (55−60 °C) and good blend-compatibility with other inorganic additives [27]. Despite PCL’s exceptional physical qualities for hard tissue engineering applications, its hydrophobicity remains its primary drawback for further development in biomedical engineering [28]. We combined PCL with calcium silicate (CS) powder, a hydrophilic ceramic biomaterial to overcome this issue. CS-based materials, including bioactive glass and calcium silicate cements, have been commonly used in hard tissue engineering applications [29,30,31]. The hydrophilic nature of CS and its ability to develop calcified apatite outer layers can enhance cellular attachment, proliferation, and differentiation; therefore, CS is a promising biomaterial for bone tissue regeneration [32,33]. It has been further reported that extracellular calcium ions from CS have significant effects on osteoblast proliferation and differentiation [9]. Most importantly, the content ratio of the silicon ions in CS-based materials can influence the adsorption of different type of ECM components, such as Col I, FN, and vitronectin, on the material surface, and can also up-regulate the signaling pathway of MAPK/ERK and MAPK/p38 [34].

According to the results of the previous study [35], we developed three-dimensional (3D) printed bioactive CS/PCL scaffolds by utilizing extrusion-based additive manufacturing technology, thus avoiding the use of any organic toxic solvents. In order to enhance the biological behavior, we coated dECM derived from MG63 cells. The extent of cellular adhesion, proliferation, and osteogenesis of these bioscaffolds were tested in vitro with human Wharton’s Jelly mesenchymal stem cells (WJMSCs) and in vivo with Wistar rats.

## 2. Results and Discussion

### 2.1. The Characterizations of CS/PCL Scaffold

**Figure 1:**
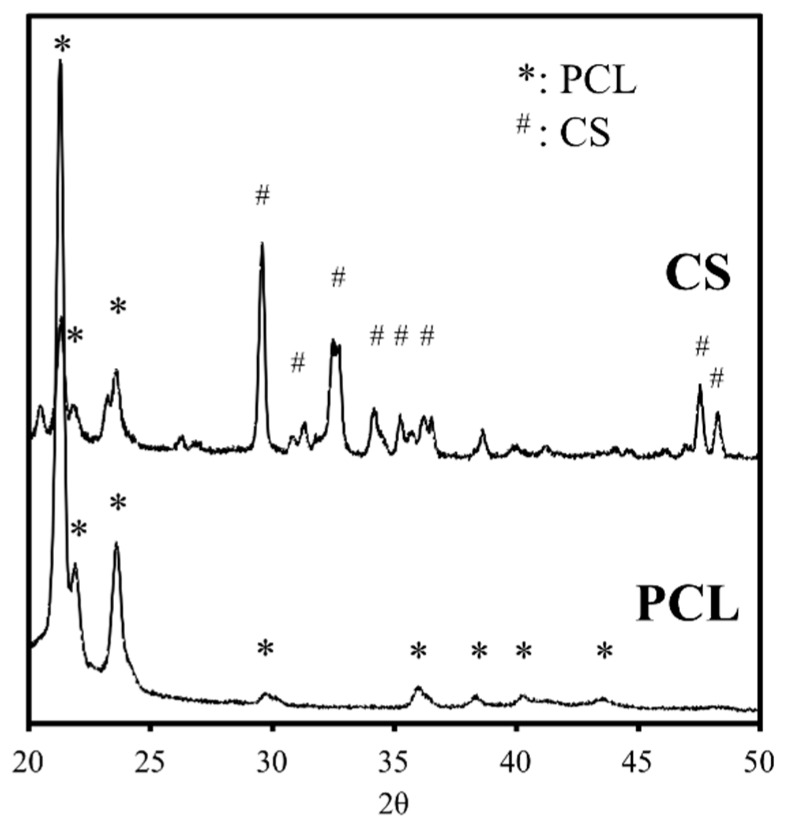
X-ray diffractometry (XRD) patterns of the polycaprolactone (PCL) scaffolds with various ratios of calcium silicate (CS). #: Characteristic peaks of CS; *: Characteristic peaks of PCL.

The XRD analysis indicated that the diffraction peaks were at 2θ = 21.44°, 22.06°, 23.76°, and 36.32°, which are characteristic peaks of PCL (Figure 1). The presence of these narrow peaks makes sense when we consider PCL as a semi-crystal polymer. The diffraction peaks at 2θ = 29.4° correspond to the calcium silicate hydrate (CSH) gel. Incompletely reacted inorganic component phases of the CS are represented at between 2θ = 32° and 34°. We confirmed that the addition of CS resulted in the lower peak intensities displayed at the PCL phases. The reduction in the intensity of the peaks is due to changes in the PCL’s micro-structural and crystallinity. In our previous studies, stress-strain analysis was performed on composites that were made out of CS and PCL in different ratios and the results indicated that scaffold’s compressive strength increases proportionally with its CS concentration [35].

**Figure 2:**
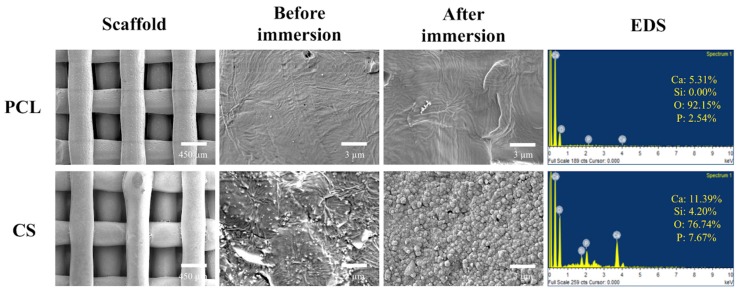
Surface scanning electron microscope (SEM) images and energy dispersive spectroscopy (EDS) assay of the scaffolds before and after immersion in DMEM.

SEM imaging was performed to observe the interconnected porous structural design, macropores, and physical changes on the scaffolds after being immersed in DMEM (Figure 2). In all of the specimens, the pore size was about 500 μm × 500 μm, which is large enough to potentially enhance bone tissue regeneration through ensuring uninterrupted vascularization network formation [36]. The magnified SEM images of the scaffold surface microstructure that are shown in Figure 2 indicate that the scaffolds with higher percentage of CS content exhibited an increased amount of precipitation of the apatite layer after DMEM immersion, which, according to previous studies, can dramatically improve osteogenesis [37]. However, the pure PCL group did not present the formation of apatite as did CS groups after 24 h of DMEM immersion. The CS surface structure was uniformly covered with spherical minerals with an average size of approximately 1 μm. The Ca/P ratios of the DMEM-immersed scaffold were 2.09 and 1.49 for PCL and CS, respectively. The Si–OH functional groups on the surface of CS materials have been demonstrated to act as nucleation centers for apatite precipitation. The Ca ions released, which possibly originated from the less-level hydration products, could significantly affect apatite growth by promoting local Ca concentration.

### 2.2. dECM Prepared from MG63

**Figure 3:**
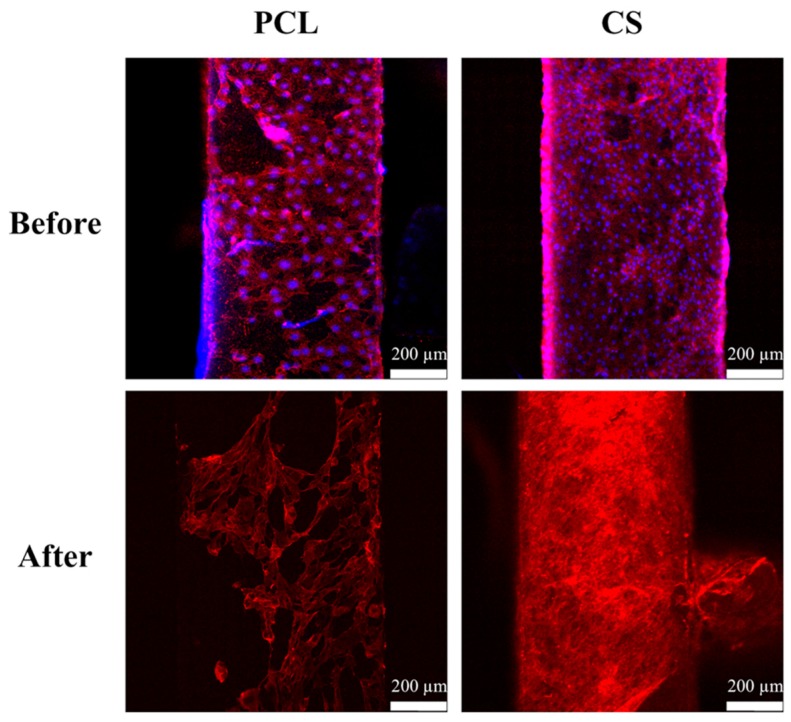
MG63 cultured and deposition of ECM from cells on the surfaces of scaffolds. F-actin cytoskeleton (red) and cell nuclei (blue) staining of MG63 cells.

Decellularization is a technique that has recently been increasingly employed, as the resulting dECM retains important protein components that could play an important role in improving cellular adhesion, proliferation, and differentiation. The decellularization process removed the intracellular content of the MG63 cells and retained the cytoskeleton framework of the cells. Figure 3 provides confocal microscopy images that show the nucleus and the cytoskeleton of the MG63 cells before and after decellularization. The amount of MG63 cell that ECM preserved after decellularization is dependent on the physical nature of the scaffolds. Scaffolds with CS content were covered with more dECM due to the hydrophilic nature that is endowed by CS; this phenomenon was also witnessed in the results of the red F-actin staining of the cytoskeleton after decellularization. The chemical decellularization procedure that was used in this study removed the intracellular content by piercing through the cellular membrane and allowed the removal of the intracellular contents [38]. Thus, during the decellularization process, the cells shrank and became more sparsely distributed than those before undergoing decellularization [5].

**Figure 4:**
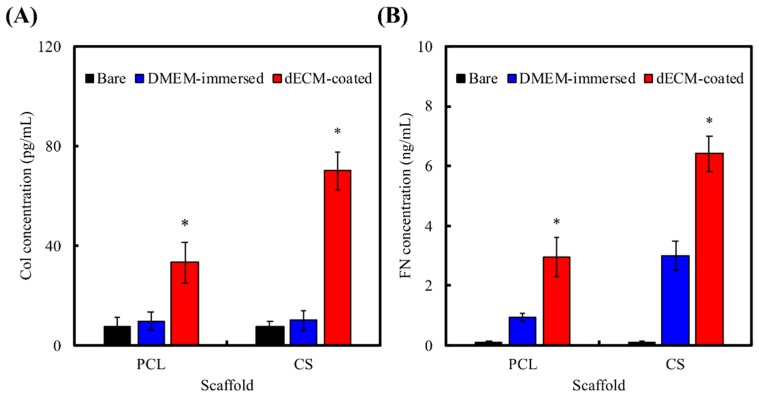
(**A**) Fibronectin (FN) and (**B**) collagen I (Col I) adsorbed on various scaffold after different treatment. “*” indicates a significant difference (*p* < 0.05) when compared to the Dulbecco’s modified Eagle medium (DMEM)-immersed group.

Figure 4A,B shows the amount of FN and Col I adsorbed on various scaffold after DMEM-immersion or dECM-coating. As shown in Figure 4A, there is a statistically significant difference (*p* < 0.05) in increased FN concentration in the DMEM-immersed scaffold group when compared to the bare scaffold group. Furthermore, FN concentration on the dECM-coated scaffolds was significantly (*p* < 0.05) higher than those of other groups. Since then, FN is one of the main component of FBS, scaffolds can experience FN adsorption upon immersion in DMEM containing FBS [6]. In contrast to the results of FN concentration, the results of Col I concentration in the DMEM-immersed scaffold group and the bare scaffold group presented no significant statistic difference (*p* < 0.05); the reason could be that Col 1 is not presented in FBS. However, there was a statistically significant increase in Col I adsorption in the dECM-coated scaffolds (*p* < 0.05), which is probably due to the hydrophilicity of the scaffolds that are endowed by CS and the existence of dECM on the surfaces of these scaffolds. These results may explain the reason why more cells adhere on to surfaces of scaffolds with CS contents, since there tends to be a direct relationship between protein (FN and Col I) adherence and CS of scaffolds. The dECM components are different across various cell types. The most common ECM components of MG63 cells are Col I, FN, decorin, and versican [39]. In our recent study, we proved that Col I and FN-ECM components preferably adsorb on the surfaces of CS-based materials [34]. Several studies have also proven that a material’s surface properties can affect the adsorption of ECM [40], including un-hydrated CS, which could support a suitable local environment for concentrating Ca and Si ions that stimulated cells to secrete ECM [34]. The intact preservation of dECM on DCS can be explained by the fact that, when compared to PCL, CS has silicon content on the surface, on which ECM of MG63 can selectively adsorb. The results in the present study were in agreement with those of the previous studies.

**Figure 5:**
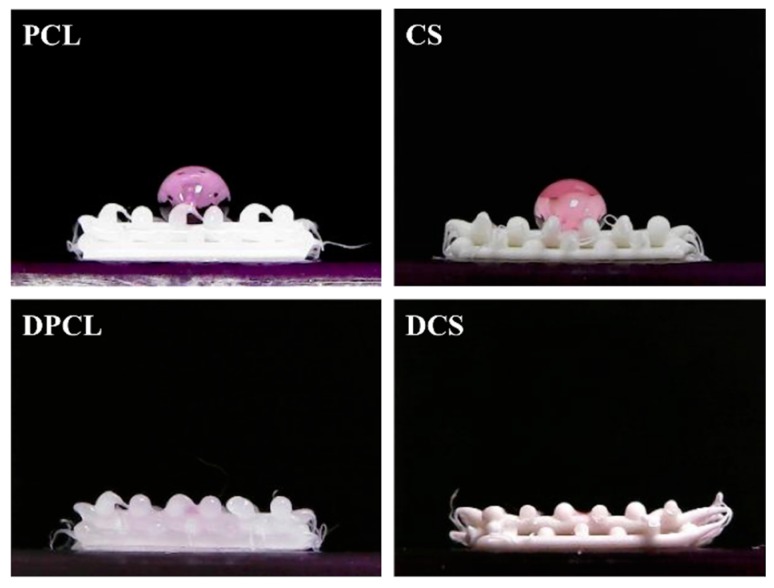
Water contact angle image of DMEM droplet on PCL and CS scaffold without/with MG63-dECM coated.

Figure 5 shows the contact angle test of the scaffolds with/without dECM coating. The contact angle decreased CS; PCL and CS exhibited contact angles of 115.3 ± 4.2° and 84.2 ± 3.7°, respectively. The CS had a significantly smaller (*p* < 0.05) angle, which coincided with the published results of another study [41]. Furthermore, the hydrophilicity of the scaffolds was further significantly enhanced after we coated dECM over the scaffolds as the DMEM infiltrated into the material right after it came into contact with the scaffolds. Cai et al. proved that the faster spreading of water could be due to the relatively larger amount of embedded hydrophilic dECM components [39]. Cellular adhesion ability can be positively influenced if it was seeded on a scaffold with a water contact angle lower than 80°. Overall, our data show that the PCL-based scaffolds were more hydrophobic and specimens that were coated with dECM were extremely hydrophilic [42].

### 2.3. Cell Adhesion and Proliferation

**Figure 6:**
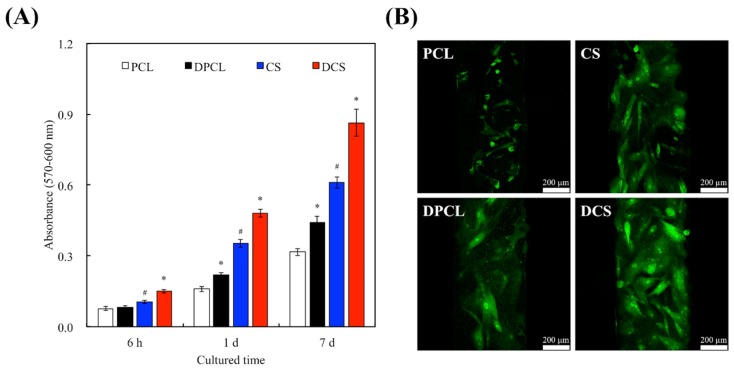
(**A**) The adhesion (6 h) and proliferation (1 day and 7 day) of Wharton’s Jelly mesenchymal stem cells (WJMSCs) cultured with scaffolds for different amounts of time. “*” indicates a significant difference (*p* < 0.05) when compared to scaffold without dECM-coated. “#” indicates a significant difference (*p* < 0.05) when compared to PCL. (**B**) Spreading and morphology of WJMSC cells on the scaffold-coated dECM after 24 h. The WJMSC were stained with an F-actin cytoskeleton (green).

Cellular adhesion is critical for cell growth, as it triggers the onset of the cell cycle, which includes proliferation and differentiation once the cells are attached onto a substrate [21]. The PrestoBlue^®^ cell viability assay was performed to examine the effects of cellular adhesion and proliferation of the WJMSCs on the scaffolds (Figure 6A). It is noteworthy that the WJMSC-adhesion absorbance results from CS scaffolds was statistically higher (*p* < 0.05) than those of the PCL scaffold. Lacking in hydrophilic characteristic of PCL led to the worst outcome in this study. The result indicated that the presence of CS was found to improve performances of adhered cells on the 3D scaffolds. Cell viability and proliferation were higher in the CS scaffolds when compared to the PCL scaffolds, thus indicating that CS could be used a bioactive material with enhanced biocompatibility [35]. Furthermore, it was previously reported that hydrophobic surfaces down regulate cellular behaviors and activities. However, CS containing scaffolds are found to be extremely hydrophilic [25]. After 6 h of WJMSC seeding, DCS exhibited significantly higher adhesion results as compared to the respective control groups that were without dECM coatings. The cellular adhesion after 6 h of seeding were found to be 1.57 times higher than their respective control groups. No significant differences in cell adhesion were found between the WJMSCs that were cultured on PCL and DPCL. Figure 6B shows that dECM upregulated the adhesion process of the WJMSCs; the amount of WJMSCs on scaffolds were greatest on DCS group. Cellular behavior and material integration of cells are usually determined by the initial adhesion process, which is also closely related to the eventual cellular proliferation, differentiation, and formation of new bone cells [25]. The ECM components (e.g., Col I) and polycations (e.g., poly-lysine) could be the reason for the enhanced efficiency of cell behavior. There are also various bioactive factors in the ECM that may act as chemoattractant to promote cell adhesion [9]. The cellular attachment result observed in this study could be due to the presence of structural proteins, signaling molecules, growth factors, and cytoskeleton framework that were retained in the ECM after decellularization.

Some studies have proved that Si ions released from biomaterials play an important role in stimulating the proliferation of primary cell [34]. When compared to their correlating control groups, DPCL and DCS increased cellular proliferation by 1.31- and 1.43-times after seven days of cell culturing, respectively. After cell adhesion and proliferation, cells can not only secrete even more ECM proteins, such as FN and Col I, but can also respond to various ECM components that help to maintain homeostasis [43]. In general, enhanced interactions between cells and ECM result in better cellular adhesion, which could trigger cell signaling cascades that up-regulate the phosphorylation of several proteins, such as focal adhesion kinase [34], providing an synergistic effect on cellular proliferation. Effective cell adhesion, proliferation, and differentiation can be witnessed under the stimulation of various surface properties of substrates [44]. Our results imply that the dECM-coating, which contributed to the bio-functionality of our CS scaffolds, is cell-friendly and allows for more uniform distribution of seeded cells. In addition, during the cell adhesion, the dECM can induce attachment protein expressions, including integrins that serve as a link between a cell’s surface membrane and the ECM; hence, the cell adhesion effect on dECM-coated CS is more enhanced than that on pure CS. The presence of more integrins is also beneficial for the induction of osteogenesis and mineralization [45]. Various studies have indicated that Si ions induce the positive regulation of cell proliferation via the MAPK/p38 and MAPK/ERK signaling pathways that are related to angiogenesis and osteogenesis, respectively [46]. Si ions can also enhance the cellular secretion of Col complexes that ultimately deposit on the substrates, accelerating adhesion process, and allowing cells to enter the cell cycle faster, which is essential for the phosphorylation of the MAPK/ERK pathways [9]. Cells require a favorable proteinaceous substrate on which cell receptors can bind to and form a cell-anchoring point with the substrate.

### 2.4. Inflammation Response

**Figure 7:**
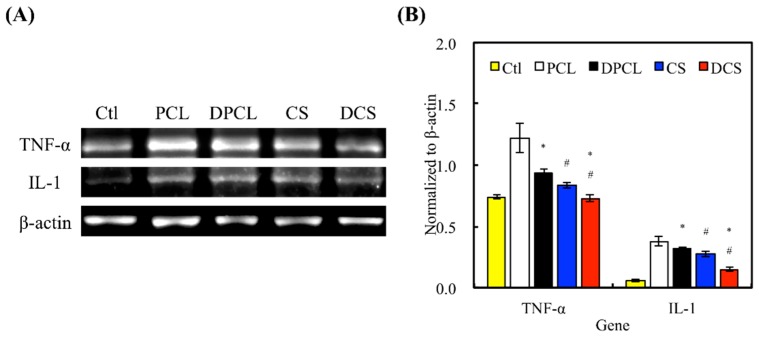
(**A**) The expression levels of inflammation-related gene (tumor necrosis factor α (TNF-α) and interleukin-1 (IL-1)) in WJMSCs cultured with various specimens via RT-PCR (β-actin served as a gene loading control). (**B**) The quantification of inflammation-related gene, respectively. Data presented as mean ± SEM, *n* = 5 for each group. “*” indicates a significant difference (*p* < 0.05) when compared to scaffold without dECM coating. “#” indicates a significant difference (*p* < 0.05) when compared to PCL. “Ctl” represented cells that grew in empty wells without any scaffolds.

In tissue engineering, good biocompatibility, which permits implantations without significant immunological rejection, is an essential characteristic of scaffolds [47]. The effectiveness of implanted materials in tissue engineering is critically dependent on the host’s immune response; therefore, favorable scaffolds should not induce graft-rejecting immune reactions that inhibit the regeneration process [47]. The evaluation of our biomaterial’s influence on the immune system in vitro is essential when completing an evaluation of biocompatibility. In this study, cytokine profiles of pro-inflammatory tumor necrosis factor α (TNF-α) and interleukin-1 (IL-1) of cellular immune responses were investigated. TNF-α plays an important role in innate and adaptive immunities and it is a pleiotropic cytokine that is associated with various inflammatory and autoimmunity responses [48]. IL-1 is a pro-inflammatory cytokine that plays a major role in the immune response of cells at the site of injuries [49]. The expression of gene encoding TNF-α and IL-1 were tested using RT-PCR after seven days of WJMSC culturing (Figure 7). The RT-PCR showed that the amount of TNF-α and IL-1 inflammation factors that were released by the WJMSCs were significantly less on CS than on PCL. Furthermore, DCS and DPCL showed significantly less evidence of inflammation when compared to CS and PCL. In our previous study, our results proved that the appropriate Si ion concentration that was released from CS materials can effectively inhibit the biological response that produces inflammatory cytokines in various cells [50]. Lu et al. found that neutrophils and macrophages will aggregate at the site of in vivo biomaterial implantations; however, fewer inflammation mediators were witnessed at the same site on the dECM scaffold implantations [5]. Another study also showed that ECM-containing hydrogel can affect innate immunity by changing the M2/M1 ratio of the host response [51]. Therefore, dECM is a good material choice for scaffold manufacturing, since it effectively mimics the structure and organization of the native tissue and reduces implant-induced immune responses; similarly, CS also has a significant effect in enhancing the biocompatibility of the scaffold, as mentioned above.

### 2.5. Osteogenesis and Mineralization

**Figure 8:**
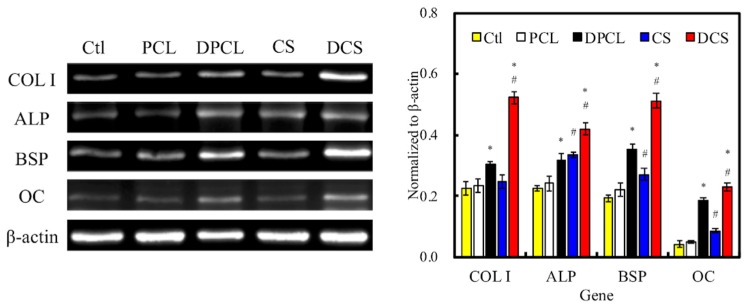
(**A**) The expression levels of osteogenic-related gene (COL I, alkaline phosphatase (ALP), bone sialoprotein (BSP), and osteocalcin (OC)) in WJMSCs cultured with various specimens for 7 days via RT-PCR (β-actin served as a gene loading control). (**B**) The quantification of inflammation-related gene, respectively. Data presented as mean ± SEM, *n* = 5 for each group. “*” indicates a significant difference (*p* < 0.05) when compared to scaffold without dECM-coated. “#” indicates a significant difference (*p* < 0.05) when compared to PCL. “Ctl” represented cells that grew in empty wells without any scaffolds.

**Figure 9:**
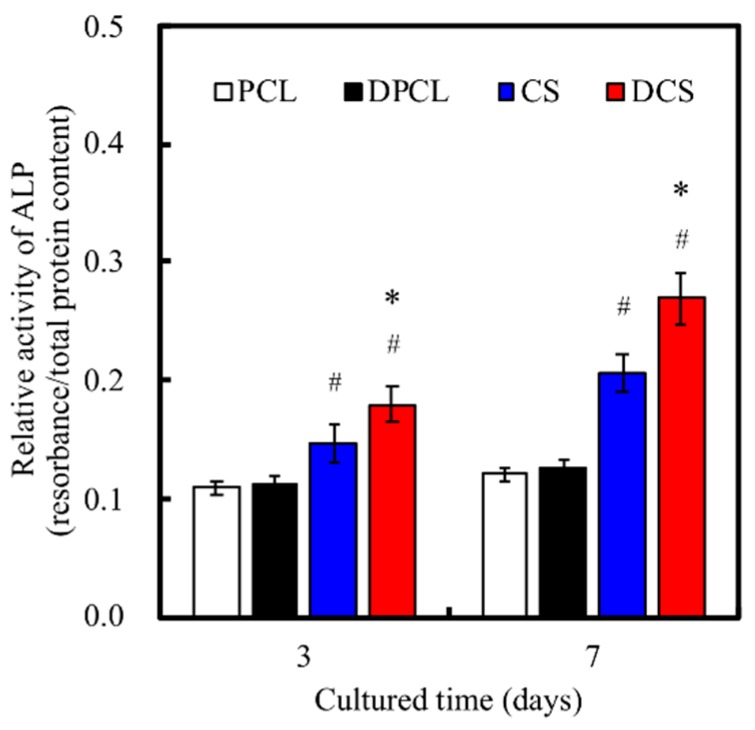
ALP activity of WJMSCs cultured on various specimens for different time periods. “*” indicates a significant difference (*p* < 0.05) when compared to scaffold without dECM-coated. “#” indicates a significant difference (*p* < 0.05) when compared to PCL.

The in vitro osteogenic-related gene expression assay (Figure 8) clearly indicated that the WJMSCs that were cultured on ECM-coated scaffolds exhibited significantly higher expression of Col I, ALP, BSP, and OC genes after seven days when compared to cells that were cultured on bare scaffolds without dECM and those on the control groups, which were represented by WJMSCs that grew in empty wells without any scaffolds. All of the normalized osteogenic biomarker readings were significantly higher in the groups with dECM (DPCL and DCS) as compared to the raw material groups (PCL and CS). The amount of Col I, BSP, and OC that were expressed in the DCS groups were at least two-fold of those expressed in CS. The Col I is exclusive to hard tissue formation, and a higher amount of collagen secretion has been associated with MAPK/ERK activation [9]. It is also important to note that DPCL had higher gene expression for Col I, BSP, and OC than CS, indicating that, perhaps, dECM plays more crucial roles than CS during the process of WJMSC osteogenesis. Nevertheless, scaffolds with CS content and dECM coating expressed more osteogenic biomarkers than scaffold without dECM modification. Additionally, these differentiation results further substantiated the idea that the ion-release kinetics of CS accelerates the process of differentiation through a faster initiation of the cell cycle due to more effective cellular adhesion and, hence, earlier induction of either the MAPK/ERK or Wnt/catenin signaling pathway [34,37]. ALP is a bio-indicator for early osteoblast differentiation and it plays an essential role in the initiation of matrix calcification. Figure 8B and Figure 9 suggest that the dECM from MG63 cells enhanced early osteogenic differentiation of WJMSC in vitro, which was possibly due to the fact that ECM includes various biological factors that induce osteogenesis differentiation. ALP activity on DCS was higher than on other groups (Figure 9); these observations are similar to our osteogenic-related gene expression results. DCS and DPCL all induced more cellular ALP secretion than did CS and PCL, respectively. Our results indicated that the MG63-secreted ECM facilitated the differentiation of WJMSCs into cells that were of osteogenic lineages. Similarly, the CS scaffold exhibited more mineral calcification and ALP expression.

**Figure 10:**
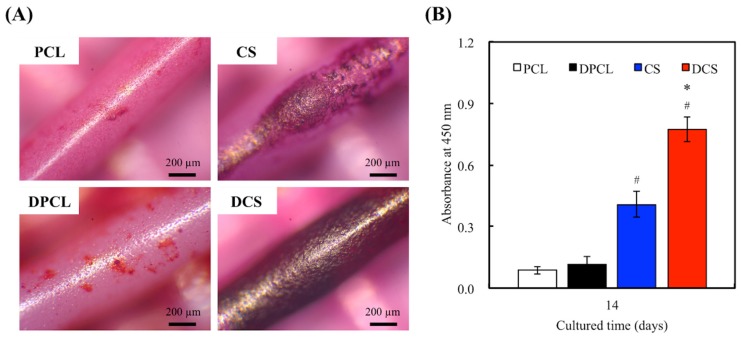
(**A**) Alizarin Red S staining and (**B**) quantification of calcium mineral deposits by WJMSCs cultured on all scaffolds for two weeks. “*” indicates a significant difference (*p* < 0.05) compared to scaffold without dECM-coated. “#” indicates a significant difference (*p* < 0.05) when compared to PCL.

Alizarin Red S stain was used to determine calcium deposition from the cells (Figure 10A). The CS scaffolds increased the amount of calcium mineral deposits after two weeks of differentiation culturing, as Alizarin Red S staining of the specimen presented a deeper shade of pink in groups with CS content. The results observed for CS were similar to those of the experiments that were performed on CS-materials in another study [29]. Figure 10B indicates the optical densities of Alizarin Red S that was extracted from the stained specimens to quantify the calcium (Ca) mineral deposits. The Ca mineral deposit levels in DPCL and DCS were 1.3- and 1.9-times higher, respectively, than the corresponding scaffolds without dECM. The Ca deposition, which is generally regulated by the bone cell, was greater on the dECM-coated scaffolds than on the bare 3D printed scaffolds. In fact, the surface of PCL also had dECM adsorption and there was not much Col I on it, so it is unable to effectively improve the efficiency of cell mineralization. On the other hand, Si ion that is released from CS can enhance MG63 secretion and ECM contained more Col I and it adsorbed on the scaffold surface. Thus, these results verify that dECM can amplify the extent of mineralization for bone regeneration [52]. Therefore, we will directly compare the bone regeneration between CS and DCS in vivo.

### 2.6. In Vivo Bone Formation in Calvarial Defects

**Figure 11:**
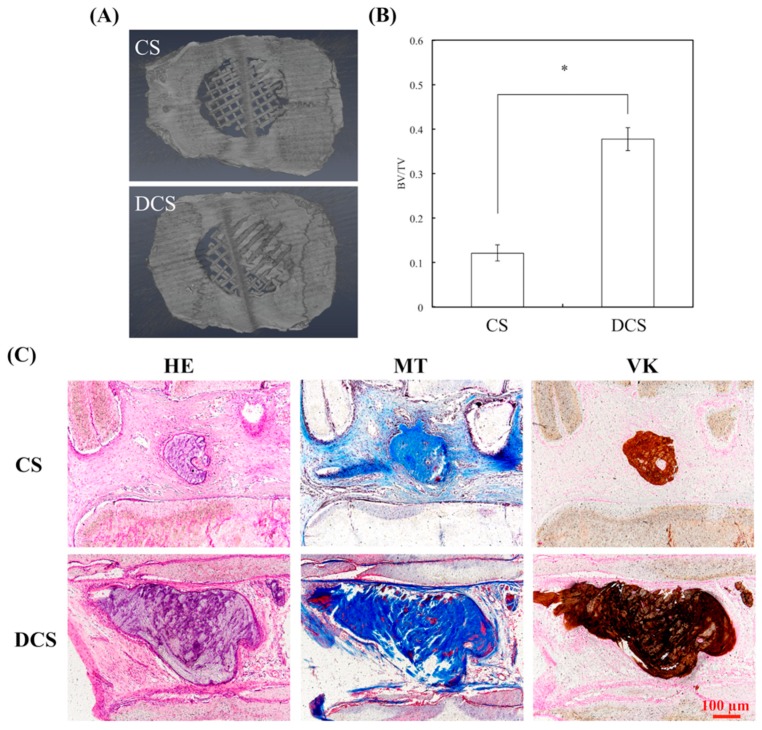
(**A**) µ-CT image showing the morphology of bone growth at fixed sized critical lesion after undergoing four-week regeneration with CS and DCS scaffolds; (**B**) Data analysis of relative bone mass volume (BV/TV) at fixed sized critical lesion after undergoing four-week regeneration with CS and DCS scaffolds; “*” indicates a significant difference (*p* < 0.05) when compared DCS to CS. (**C**) Left: hematoxylin and eosin (HE) stain; Middle: Masson’s trichrome (MT) stain; Right: Von Kossa (VK) stain of regenerated bone mass after four weeks of in vivo experiment.

To further testify the effect of dECM on bone tissue regeneration, in vivo experiments were performed on adult white rats. The µ-CT images (Figure 11A) showed that much more new bone formation was observed in the DCS specimens as compared to the CS groups four weeks after implantation. In addition, the morphometrical analysis (Figure 11B) indicated that a significantly greater BV/TV value is seen in the DCS group (37.75% ± 2.58%) as compared to the CS (12.18% ± 1.81%) after four weeks of implantation. Subsequently, the results of the undecalcified specimens stained by the hematoxylin and eosin (HE) stain, Von Kossa (VK) stain, and Masson’s trichrome (MT) stain (Figure 11C). From the HE staining images, there were no apparent inflammation cells identified in CS and DCS. In addition, there was no fibrous tissue that was detected in the defect areas in the DCS group, and a large amount of Col extracellular matrix had filled the spaces between the materials. It was known that VK staining is highly expressed in newly formed bone tissue. Particularly, DCS showed more VK staining that ascribed to the dECM enhances the bone regeneration ability of CS scaffolds for the critical size of bone defects. These stainings that exhibited richer bone formation in the DCS groups represent the newly formed bone that was detected in the periphery and center of the bone defect. In the critical-size calvaria defect, bone tissue regeneration appears to be promoted by the dECM that was coated on 3D printed scaffold, through providing an appropriate microenvironment. Furthermore, the dECM-coated scaffolds promoted a significant increase in the mineralized tissue area in a microenvironment, when compared to bare ceramic scaffold. The cell-generated ECM has previously been illustrated to promote osteogenesis and improve engraftment in animal models [53]. Therefore, the in vivo bone tissue regeneration capability of the bioscaffolds that were supplemented with dECM could be considered for clinical use in the future.

## 3. Materials and Methods

### 3.1. CS Powder Preparation

The method that is used here for the preparation of the CS powder has been described elsewhere [54]. In brief, reagent grade CaO (Sigma-Aldrich, St. Louis, MO, USA), SiO_2_ (High Pure Chemicals, Saitama, Japan), and Al_2_O_3_ (Sigma-Aldrich) powders were used as matrix materials (composition: 70% CaO, 25% SiO_2_, and 5% Al_2_O_3_). The oxide mixtures were then sintered at 1400 °C for 2 h using a high-temperature furnace. The CS powder was ball-milled in 99.5% ethanol in a centrifugal ball mill (S 100, Retsch, Hann, Germany) for 6 h and then dried at 100 °C overnight.

### 3.2. Preparation of the CS/PCL Paste

CS powder was immersed in 99.5% ethanol and stirred at 400 rpm for 6 h. The homogenously dissolved CS in ethanol was slowly added into molten PCL beads (Mw = 43,000–50,000, Polysciences, Warrington, PA, USA) and stirred with a metal rod at 200 °C until the CS-ethanol solution was evenly mixed with the PCL to create a CS/PCL paste; simultaneously, ethanol in the paste would evaporate due to its relatively low boiling point. After mixing, the paste was left in the oven at 85 °C for 24 h. In this study, the specimens coded as ‘PCL’ and ‘CS’ represent the specimens contain 100% PCL and 60% CS/40% PCL, respectively.

### 3.3. Scaffold Fabrication

The 3D scaffolds were fabricated with a BioScaffolder (BioScaffolder 3.1, GeSiM, Großerkmannsdorf, Germany) has been described elsewhere [35]. The printable paste was transferred into the printing cartridge and pre-heated. Subsequently, the paste was heated to 80 °C and layered with a nozzle (20G needle) under 450 kPa of extrusion pressure at a printing speed of 2 mm/s. The scaffold measured 6.5 mm × 6.5 mm × 1.4 mm. The strut, with a width of 500 μm, were printed in parallel with a gap of 500 μm between struts. Subsequent layers were printed above the previous layer, so that the struts of the current layer were 90° to those of the underlying layer, forming 500 μm × 500 μm pores. After printing, the scaffold was left to dry at room temperature for 2 h.

### 3.4. Phase Compositions and Morphology

The phase composition of the scaffold was analyzed using X-ray diffractometry (XRD; Bruker D8 SSS, Karlsruhe, Germany) performed at 30 kV, 30 mA, and scanning speed of 1°/min. The morphology of the scaffold was coated with gold and then examined under a scanning electron microscope (SEM; JSM-6700F, JEOL, Tokyo, Japan) that operated in the lower secondary electron image (LEI) mode at an accelerating voltage of 3 kV. In order to analyzed the bioactivity of scaffolds, the specimens were soaked in Dulbecco’s modified Eagle medium (DMEM, Invitrogen, Waltham, CA, USA) containing 10% FBS. After immersion for one day, the scaffolds were removed from DMEM, and the Ca, Si, O, and P on the scaffold surface were investigated by energy dispersive spectroscopy (EDS; JSM-6700F, JEOL).

### 3.5. Decellularized ECM Coating and Characterization

Before cell seeding, the scaffolds were immersed in 75% ethanol under UV light for 1 h. Subsequently, the scaffolds were placed in a 48-well plate in which MG63 cells with a cell density of 5 × 10^4^ cells were seeded onto the scaffold. The MG63 cells were cultured on the scaffolds for five days with DMEM containing 10% FBS and the medium was changed once every two days. The decellularization method was previously described in other published studies [38]. In short, the cells were treated with the decellularization solution, consisting of 0.5% Triton X-100 and 20 mM NH_4_OH (Sigma-Aldrich) in phosphate buffered saline (PBS, Invitrogen) at a pH = 7.4 for 5 min at 37 °C and subsequently washed with PBS and treated with 12.5 U/mL DNase I (Sigma-Aldrich) for 1 h at 37 °C. After the decellularization process, the scaffolds were cleaned with PBS 10 min on a shaker three times and then stored at 4 °C until use. In this study, the specimen codes ‘DPCL’ and ‘DCS’ represented the decellularized ECM that was coated on specimen PCL and CS, respectively. In addition, the dECM-coated scaffold was fixed with 4% paraformaldehyde for 10 min and then washed with PBS. Afterwards, the specimens were immersed in phalloidin conjugated to Alexa Fluor 594 (1:500 dilution in PBS, Invitrogen) and DAPI (1 µg/mL, Invitrogen) to obtain an F-actin cytoskeleton and nuclei fluorescent signals, respectively. The fluorescent stains on the non-decellularized MG63 cells on scaffolds were performed using the method that is described above except that 0.1% Triton X-100 was treated for 10 min at room temperature prior to the administration of the stains. The staining results of the MG63 cells on the scaffolds were observed under a Leica TCS SP8 X white light laser confocal microscope (Leica Microsystems GmbH, Wetzlar, Hessen, Germany). The water contact angle for each specimen was determined at room temperature. Briefly, the specimens were placed on the top of a stainless-steel base; a drop of MilliQ water (10 μL) was placed on the surface of the specimens and an image was taken with a camera after 20 s had elapsed. The resulting images were analyzed using ImageJ (National Institutes of Health, Bethesda, MD, USA) to determine the water contact angle.

### 3.6. ELISA Analysis

ELISA was used to analyze Col I and FN concentration in pure scaffolds, DMEM-immersed scaffolds, and dECM-coated scaffolds. An enzyme-linked immunosorbent assay kit (Invitrogen) was used to consider the concentration levels of Col I and FN by following the instructions in the manufacturer’s manual. Subsequently, the levels of Col I and FN were determined by correlation with a standard curve. Six independent experimental analyzes were performed for each specimen.

### 3.7. Cell Adhesion and Proliferation

The human Wharton’s Jelly mesenchymal stem cells (WJMSCs) were obtained from the Bioresource Collection and Research Center (BCRC, Hsin-Chu, Taiwan) and then grown in a mesenchymal stem cell medium (#7501, Sciencell, Carlsbad, CA, USA) to passage 3–6. The WJMSCs were directly cultured on the scaffolds at a density of 5 × 10^4^ cells in a 48-well plate and incubated at 37 °C in a 5% CO_2_ atmosphere for various durations (Adhesion: 3 h; Proliferation: one and seven days). After different culturing, the cell viability was evaluated using the PrestoBlue^®^ (Invitrogen) assay. At the end of the culture period, the DMEM was removed and the wells were washed twice with cold PBS. Subsequently, each well with a specimen was filled with PrestoBlue^®^ and fresh DMEM at a ratio of 1:9 and incubated at 37 °C for 60 min. The resulting solution in each well was then transferred to a new 96-well plate and the optical density (OD) of the solutions was measured using Tecan Infinite 200^®^ PRO microplate reader (Tecan, Männedorf, Switzerland) at 570 nm with a reference wavelength of 600 nm. The results were obtained in triplicate from three separate experiments.

### 3.8. Reverse Transcription Polymerase Chain Reaction

The inflammation-related (tumor necrosis factor-alpha (*TNF-α*) and interleukin-1 (*IL-1*)) and bone-related gene (*Col I*, alkaline phosphatase (*ALP*), bone sialoprotein (*BSP*), and osteocalcin (*OC*)) of the WJMSCs were detected after seven days of culturing. The gene expression level was normalized to the β-actin for each group. Total RNA of all five groups was extracted using TRIzol reagent (Invitrogen) after seven days and then analyzed using RT-PCR. Total RNA (500 ng) was used for the synthesis of complementary DNA using a cDNA Synthesis Kit (GeneDireX, Las Vegas, NV, USA) following the manufacturer’s instructions. RT-PCR primers (Table 1) were designed based on cDNA sequences from the NCBI Sequence database. Each PCR product was analyzed using 2% agarose (in TAE buffer) gel electrophoresis and then visualized by Novel Juice (GeneDireX) staining. The stained bands were photographed using a digital camera. Each sample was performed in triplicate.

### 3.9. Osteogenesis Assay

The level of ALP activity was determined after the cells were cultured in an osteogenic medium (StemPro™ osteogenesis differentiation kit, Invitrogen) for three and seven days, respectively. The process was as follows: the cells were lysed from discs using 0.2% NP-40 and centrifuged for 10 min at 2000 rpm after washing with PBS. ALP activity was determined using p-nitrophenyl phosphate (pNPP, Sigma) as the substrate. Each sample was mixed with pNPP in a 1 M diethanolamine buffer for 15 min, after which the reaction was stopped by the addition of 5 N NaOH and quantified by absorbance at 405 nm. All of the experiments were done in triplicate. The analyzed blank disks were treated as controls.

### 3.10. Alizarin Red S Stain

The accumulated calcium deposition on the WJMSCs after 14 days of culturing on scaffolds in osteogenic differentiation medium was analyzed using Alizarin Red S staining, as developed in a previous study [9]. In brief, the specimens were fixed with 4% paraformaldehyde (Sigma-Aldrich) for 15 min and then incubated in 0.5% Alizarin Red S (Sigma-Aldrich) at a pH of 4.0 for 15 min at room temperature. After this, the cells were washed with PBS and photographs were taken using the BX53 Olympus fluorescence microscope (Olympus, Tokyo, Japan) at a 200× magnification. The Alizarin Red was also quantified using a solution of 20% methanol and 10% acetic acid in water. After 15 min, the liquid was transferred to a 96-well and the quantity of Alizarin Red was determined using a spectrophotometer at 450 nm.

### 3.11. Implantation in Rat Calvaria Model

All in vivo experimental protocols and statements confirm that all of the methods were carried out in accordance with relevant guidelines and regulations. The statement to confirm that all experimental protocols were approved by the Ethical Committee for Animal Experiments of China Medical University, Taichung, Taiwan. Six-week-old Wistar rats are raised in individual cages in an animal room that was maintained at 22 °C. For assessing the bone regeneration in a bone regeneration model, the CS and DCS scaffolds are implanted on 6-mm critical calvarias defects, which is a defect of a size that does not spontaneously heal without intervention. The rats were positioned in a stereotaxic frame and then immobilized during surgery. The hair over the skull of the animals was shaved and the underlying skin was aseptically prepared using chlorohexidine scrub. To reach the calvarium, a full-thickness incision was executed on the skin along the midline from the nasofrontal to occipital region and then the subcutaneous tissue was removed by sharp dissection. The underlying periosteum was accurately incised and elevated to obtain sufficient exposure for the trephine. A trephine with an outer diameter of 6 mm was used to remove bone from the middle of the dorsal calvarium. The scaffolds of various groups that were in this study were then implanted at the site of 6-mm critical lesions. Lastly, the periosteum and the subcutaneous tissue were sequentially closed with sutures. The animals are kept on a surgical bed until they awoke and had free access to food and water thereafter. Four weeks post-implantation, the rats were sacrificed by CO_2_ asphyxiation and the calvaria bone specimens of the rats were harvested and fixed in 10% formalin.

### 3.12. Micro-Computed Tomography

Images of all in vivo specimens were taken with a high 360° spatial resolution micro-computed tomography (µ-CT, SkyScan 1076, Skyscan Inc., Kontich, Belgium), which is equipped with an X-ray CCD camera of 1.4 M. The maximum tube current is 0.2 mA, maximum tube voltage is 160 kV, and the focus size is 1 mm. For each sample images, 70-V tube voltage and 70-A current were maintained in the X-ray tube. For the scanning of whole calvaria, 400 micro-tomographic slices of every sample with a slice increment of 30 mm were taken. The micro-CT slices were processed using 3Di-Cat for thresholding that were then used to create 3D models for visualization and quantitative histomorphometric analysis. The SkyScan software was used to recognize new bony tissue and analyze the bone volume per tissue volume (BV/TV) in the 3D model.

### 3.13. Histological Staining

After the retrieval of the in vivo samples, they are fixed in 10% fomalin for 48 h, rinsed with PBS several times, without decalcified, embedded in OCT (KMA-0100-00A, CellPath Ltd., Newtown, Wales, UK), and sectioned (6-mm thick). After which, 6 μm longitudinal sections were prepared per specimen using a sawing microtome technique. The sections were prepared and stained with hematoxylin and eosin stain kit, modified Masson’s Tricrome stain kit (ScyTek Lab., West Logan, UT, USA), and Von Kossa kit (ScyTek), according to the manufacturer’s instructions. Trichrome stain in blue was used for the identification of collagen. Von Kossa staining in red was used to observe the difference between the osteoid tissue and the calcified bone. Sections were examined using the BX53 Olympus fluorescence microscope at 200× magnification.

### 3.14. Statistical Analyses

A one-way analysis of the variance statistical data was used to evaluate the significance of the differences between the means in the measured data. A Scheffe’s multiple comparison test was used to determine the significance of the deviations in the data for each specimen. In all cases, the results were considered to be statistically significant with a *p* value <0.05.

## 4. Conclusions

The organic–inorganic hybrid scaffolds with 3D connected porosity that was constituted by CS and PCL was manufactured via 3D printing. Subsequently, the scaffolds were developed to coat dECM over the scaffolds through cell culturing and the decellularization of bone cells. The inclusion of CS dramatically altered the surface characteristics of the bioscaffold by making the surface more hydrophilic. Hence, the scaffolds with CS content ratios were covered with more dECM after decellularization due to the hydrophilic characteristic that was endowed by the CS. The dECM-coated CS scaffolds significantly enhanced WJMSC adhesion, proliferation, and differentiation. Moreover, we also proved that the CS scaffolds with dECM coating could regulate the cellular immune response by decreasing the expression of pro-inflammatory cytokines. When compared to scaffolds without dECM, the dECM-coated CS scaffolds significantly promoted Ca deposition of WJMSCs. The in vivo experiments also showed the promising tissue regenerative potential of the composite that was developed in this study. With these results and our previous study regarding the physical characterization of CS, we believe that the decellularization technology, combined with 3D printed scaffolds, can be extensively applied in various tissue engineering. In the future, 3D printed scaffolds with decellularized matrices could be used as tissue regeneration models in bone tissue engineering by incorporating biocompatible synthetic materials with a natural dECM microenvironment that contains growth factors and structural proteins of matching distribution and composition for more efficient cellular behavior and optimal regenerative capacity.

## Figures and Tables

**Table 1:** Primer pairs used in this study.

Gene	Sequence
*TNF-α*	Forward: 5′-CCCAGGCAGTCAGATCATCTTC-3′
	Reverse: 5′-AGCTGCCCCTCAGCTTGA-3′
*IL-1*	Forward: 5′-AGTCAGCTCTCTCCTTTCAGG-3′
	Reverse: 5′-CTTGCCCCCTTTGAATAAAT-3′
*Col I*	Forward: 5′-CCATGTGAAATTGTCTCCCA-3′
	Reverse: 5′-GGGGCAAGACAGTGATTGAA-3′
*ALP*	Forward: 5′-TAGTTCCTGGTACCTCTGCTCC-3′
	Reverse: 5′-CAGTTTCCTTTCCTGAATACCG-3′
*BSP*	Forward: 5′- TCACCTGTGCCATACCAGTTAA-3′
	Reverse: 5′- TGAGATGGGTCAGGGTTTAGC-3′
*OC*	Forward: 5′- GTGCAGAGTCCAGCAAAGGT-3′
	Reverse: 5′- CGATAGGCCTCCTGAAAGC-3′
*β-actin*	Forward: 5′-AGAGCTACGAGCTGCCTGAC-3′
	Reverse: 5′-AGCACTGTGTTGGCGTACAG-3′
